# Tunneling Nanotubes Facilitate Intercellular Protein Transfer and Cell Networks Function

**DOI:** 10.3389/fcell.2022.915117

**Published:** 2022-07-12

**Authors:** Laura Turos-Korgul, Marta Dorota Kolba, Piotr Chroscicki, Aleksandra Zieminska, Katarzyna Piwocka

**Affiliations:** Laboratory of Cytometry, Nencki Institute of Experimental Biology, Polish Academy of Sciences, Warsaw, Poland

**Keywords:** intercellular protein transfer, proteome, cellular network, tunneling nanotubes (TNT), SILAC mass spectrometry, codeIT, cancer microenvironment

## Abstract

The past decade witnessed a huge interest in the communication machinery called tunneling nanotubes (TNTs) which is a novel, contact-dependent type of intercellular protein transfer (IPT). As the IPT phenomenon plays a particular role in the cross-talk between cells, including cancer cells as well as in the immune and nervous systems, it therefore participates in remodeling of the cellular networks. The following review focuses on the placing the role of tunneling nanotube-mediated protein transfer between distant cells. Firstly, we describe different screening methods used to study IPT including tunneling nanotubes. Further, we present various examples of TNT-mediated protein transfer in the immune system, cancer microenvironment and in the nervous system, with particular attention to the methods used to verify the transfer of individual proteins.

## Introduction

The intercellular communication has crucial impact on proper functioning of tissues and organisms. Until recently, major part of the research on intercellular communication delved into mechanisms independent on direct contact between cells, namely communication through secreted factors, e.g., cytokines, chemokines or a plethora of extracellular vesicles. The direct contact-dependent mechanisms remained less studied, with the sole exception of gap junctions which enable the transfer of small molecules. However, in recent years, more data point to the fact that also larger molecules and organelles can be transported between cells in a contact-dependent manner. One of the new possible routes enabling this kind of transport, are mediated by tunneling nanotubes, shortly TNTs. TNTs are membranous channels, connecting two or more distant cells which are able to transport different types of cargo, including vesicles (Zhu et al., 2015), individual proteins (Pasquier et al., 2012; Desir et al., 2019; Kretschmer et al., 2019) and mitochondria (Spees et al., 2006; Pasquier et al., 2013; Jackson et al., 2016; Vignais et al., 2017; Saha et al., 2022). These structures are actin-rich, have diameters ranging from 50 to 200 nm and 30 µm mean length. It is also commonly accepted that TNTs are straight conduits hovering above the substratum (Önfelt et al., 2004; Korenkova et al., 2020; Dagar et al., 2021). There is a growing interest to investigate the precise mechanisms governing these types of interactions. In this review, we discuss several methods used to study intercellular protein transport in general. Furthermore, we review the role of tunneling nanotubes in this process and various approaches used to confirm the transport of particular molecules between cells through TNTs.

## The Phenomenon of Intercellular Protein Transfer—The Novel Perspective of Cellular Network

Generally, the processes of intercellular transport of large molecules are the most vastly reported in immune cells. These studies in the major part relate to the intercellular transfer of membrane-associated proteins, including MHC proteins, co-stimulatory proteins, NK-cell receptors for MHC class I protein, polio-virus receptor (CD155), membrane-associated antigens or antigen-specific BCR (Davis, 2007; Ahmed and Xiang, 2011). The list of possible mechanisms of this transport consists internalization and recycling pathway, dissociation-associated pathway, exosome uptake, exocytosis or more specialized secretory pathways, the enzymatic cleavage of cell-surface proteins, trogocytosis, local membrane fusion and membrane nanotube formation (Davis, 2007; Ahmed and Xiang, 2011). An increasing body of evidence both *in vitro* and *in vivo* indicates that intercellular protein transfer (IPT) is a common phenomenon, at least in the immune system (Davis, 2007; Ahmed and Xiang, 2011).

To verify whether IPT is a general phenomenon, Niu and colleagues created a mathematical formula describing IPT and experimentally verified their model predictions (Niu et al., 2009). In a 2-day long confluent co-culture of donor and acceptor cells they found, that the transfer of three different membrane proteins was bidirectional and direct contact-dependent. The transfer efficiency varied as a function of their lateral membrane mobility, related to the molecular mass, membrane fluidity and the ratio between donor and acceptor cells. Cell-cell adhesion enhanced the membrane protein transfer, therefore authors supposed that the underlying mechanism was based on transient local plasma membrane fusions. However, they did not exclude the possibility of the involvement of tunneling nanotubes in this process. In general, authors demonstrated that IPT is not restricted to a few types of proteins and occurs between multiple cell types. It must be taken into account that the possible universality of IPT challenges the classic theories of cell autonomy.

### “No Cell Is an Island” Perspective

Rechavi et al. (2009) proposed that DNA content of a cell can be considered as the “hardware,” whereas the transcriptome and proteome—as the “personality” of a cell (Rechavi et al., 2009). In this regard, the “personality” of each cell is constantly shared with other interacting cells. Therefore, cells cannot be more considered as unchangeable units of life but rather—units of life which continuously “become” what they are supposed to be in a specific situation. Although this perspective makes experimental biology more complex, it is probably closer to the physiology of living tissues and allows a holistic view on the functionality of a tissue. So far, only the neuronal system was studied with this perspective. It is commonly accepted that, although the brain consists of separate cells, the memory as well as other functions are based on intercellular contacts between neurons through synapses (Rechavi et al., 2009). IPT, as mentioned above, has several proven functional implications on biological processes, such as immune responses (Davis, 2007; Ahmed and Xiang, 2011) and presumably enables a fast modification of behaviour of large groups of cells without the need to change their gene expression profile (Niu et al., 2009). In general, research on IPT, including TNT-mediated transfer, shifts the focus on systemic level of tissue functionality. However, to achieve a more global characterization of all the proteins that transfer, there is a need for the development of high throughput technologies facilitating the identification of these groups of proteins.

## Methodology for Intercellular Protein Transfer Research

### Trans-SILAC

**FIGURE 1:**
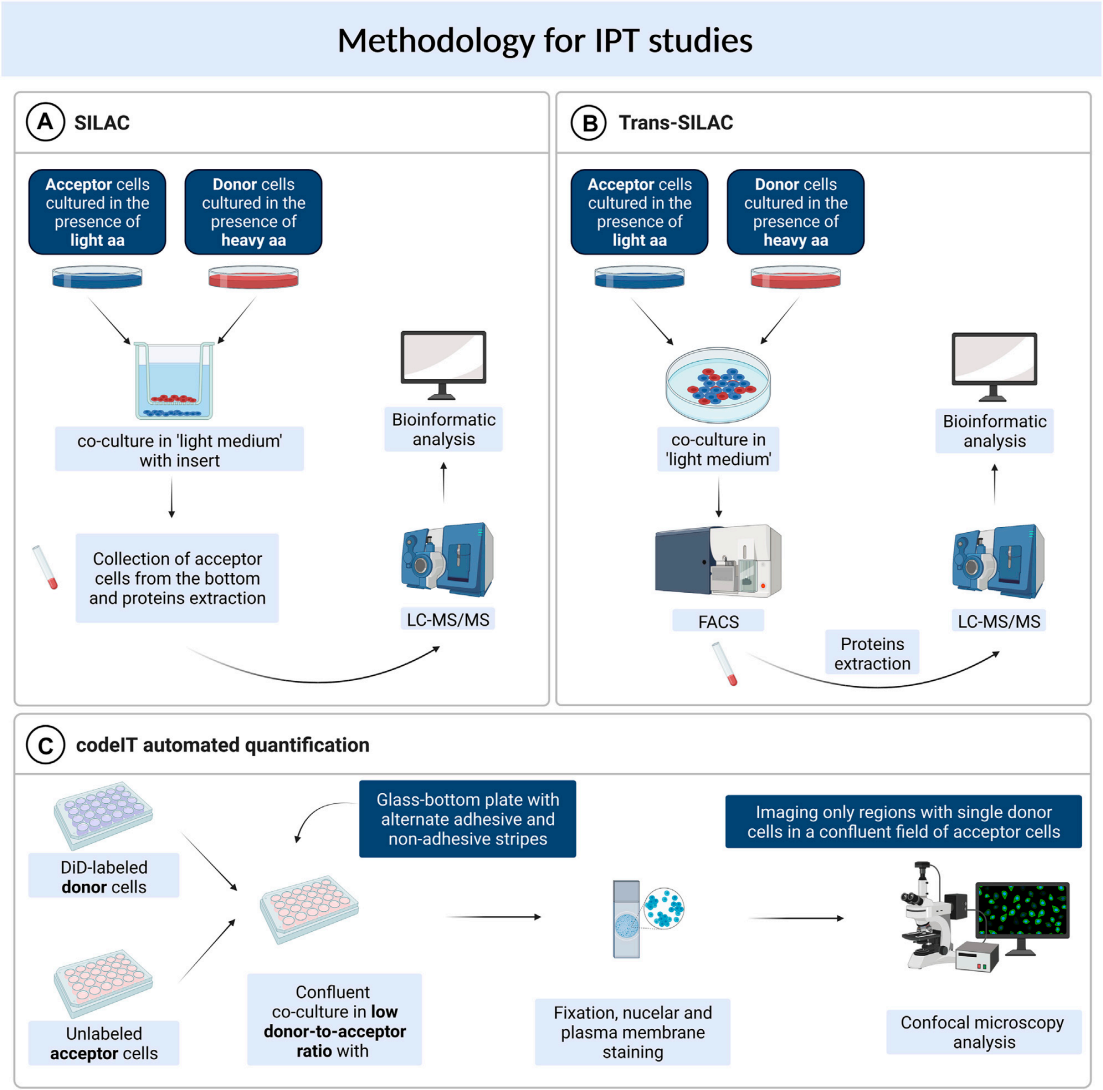
Methods used for studying intercellular protein transfer. **(A)** SILAC. **(B)** Trans-SILAC. **(C)** codeIT automated quantification.

The trans-SILAC technique is a method to identify the full repertoire of transferred proteins and therefore demark the non-cell-autonomous proteome (Rechavi et al., 2009). This technique derives from SILAC (stable isotope labelling by amino acids in cell culture), combines it with FACS (fluorescence-activated cell sorting) and provides bioinformatic tools to facilitate the identification of rare heavy proteins in a large pool of light proteins within the proteome of acceptor cells. In short, this method relies on > 98% enrichment of donor cells’ proteome in heavy isotopologues of amino acids of choice and subsequent co-culture with acceptor cells, previously cultured in the presence of light amino acids. Further on, acceptor cells get sorted by FACS on the basis of a fluorescent cytoplasmic marker (if present) or on the basis of plasma membrane markers. To additionally narrow the scope of search, a chosen subpopulation of acceptor cells might be sorted e.g. cells which received a chosen fluorescent cargo. Finally, “heavy” proteins are identified in acceptor cells by bioinformatic tools—these are the proteins which were synthesised by donor cells and transferred to acceptor cells in the scope of IPT (Figure 1). For the first time, the trans-SILAC technique was successfully used to identify sets of proteins transferred from B cells to NK cells in a direct contact-dependent and actin-dependent manner (Rechavi et al., 2009). Identified proteins grouped into functional protein networks, e.g., related to “cancer, immunological disease, and hematological disease,” identified by the network explorer feature of the Ingenuity Pathways Analysis (IPA) platform. Importantly, authors highlighted that this is an exemplary presentation of the utility of the trans-SILAC method which can be used for versatile purposes: it is available for the study of various cell types, the conditions and the time of co-culture can be specifically chosen. Moreover, one can track the direct contact-dependent or -independent IPT, as well as IPT correlated with the transfer of a given cargo or a protein of interest. No specialized instruments are required, the software created for the analysis of the LC-MS/MS data has been reposited with the original paper and is available on-line. Another use of trans-SILAC technique reported in the literature enabled the discovery that senescent cells communicate through direct contact-dependent IPT with NK cells (Biran et al., 2015). As previous reports focused on the secretory phenotype of senescent cells, the direct-contact dependent communication was a novelty in this regard. Authors found that among 47 proteins transferred from donor to NK cells, 90% transferred exclusively from cells with induced senescence, with no mass restriction for the transfer. They specifically enriched three biological processes, namely glycolysis, regulation of the actin cytoskeleton, and antigen processing and presentation (Gene Ontology analysis). What is more, the IPT led to increased NK cell activation and cytotoxicity towards senescent cells. In spite of the fact that the majority of IPT correlated with the protein abundance in donor cells, 12.5% of the identified proteins transferred independently of protein abundance in donor cells. It indicates that IPT is not a passive process but can be actively regulated.

### SILAC

Parallely, a similar concept was used to identify IPT that was independent on direct contact, with the use of the sole SILAC method (Li et al., 2010). In this study, murine lung cells previously grown in “heavy” media and irradiated, were co-cultured in a transwell system with murine bone marrow cells. After 48 h, acceptor cells were collected from the bottom chamber of the transwell and analysed by LC-MS/MS for the presence of “heavy” proteins (Figure 1). A set of seven proteins was transferred, including the retinoblastoma-binding protein 7 (RBBP7), however, the irradiation step was critical for IPT to occur. Authors found that irradiation injury of lung cells led to secretion of proteins that have not been previously regarded as secreted. These findings further highlight the biological relevance of IPT.

### Contact-Dependent Intercellular Transfer Automated Quantification

Another high throughput technique available for the study of intercellular transfer, including TNT-mediated transfer, is not restricted to proteins and can be used to identify and quantify regulators and cargo of contact-dependent intercellular transfer (termed: codeIT) (Frei et al., 2015). This method is a microscopy-based screening (Figure 1), and can be assigned also to individual donor cells, quantified by confocal microscopy and image analysis in 2D or 3D, therefore preserving spatial information. Importantly, codeIT is suited for any fluorescently labelled molecule or structure, including pathogens. It was used for the identification of regulators of transfer of endocytic vesicles labelled with lipophylic VybrantDye DiD. For the identification of regulators of the codeIT process, one can use siRNA-based screen. To exclude the possibility of intercellular transfer through the shared medium, the low donor-to-acceptor ratio should be used (in the range 1:100–1:400). To increase transfer with such a limited number of donors, the confluent co-culture is recommended. In this set-up (extracellular vesicles transfer), DiD is a specific dye and marker for codeIT. The study revealed that majority of donor cells transferred cellular vesicles in a direct contact-dependent manner. DiD transfer intensity followed a normal distribution. Additionally, the dotted pattern of the transfer as well as a correlation between the intensity of signal and the volume (sum of voxels) excluded the suspicion of dye diffusion and supported the transfer of DiD-labelled “packages”. Observed transfer was dependent on F-actin and serum components. A screen of 36 gene candidates revealed several regulators of codeIT, including Myo10, Cdc42 and several Rab proteins.

## There Is a Need for Hit Validation

It is important to note that high throughput techniques described above require further steps to validate the transfer of the identified proteins with supplementary methods. For example, the putative regulators of codeIT, identified by siRNA-based silencing, were further overexpressed as EGFP-tagged proteins, including truncated proteins and point-mutants, and searched for their influence on contact-dependent transfer of the studied vesicles with flow cytometry methods. Therefore the described assay offers a possibility of differentiating the role of candidate protein separately in donor and acceptor cells. In regard to SILAC-based methods, the proteins identified as transferred can be further validated by Western blotting, flow cytometry or other approaches. For example, the transfer of proteins from murine lung cells identified by SILAC, was validated by studying the expression levels of the proteins of interest in acceptor cells by Western blotting (Li et al., 2010). However, this method of hit validation is limited to proteins transferred in significant amounts. Additionally, to exclude the possibility of increased transcription of the relevant genes, a Real-Time PCR was performed. Finally, to ensure that proteins are transferred into the cytosol of the cell, a trypsin digestion to destroy the proteins adsorbed to outer membrane followed by Western blotting was performed. This further supported the IPT hits identified by SILAC. The protein transfer can be also validated by flow cytometry, which has been used to verify the transfer of 17 selected proteins between B cells and NK cells, (Rechavi et al., 2009). Cytosolic proteins were expressed as EGFP-tagged proteins in donor cells, whereas membrane-associated proteins were identified by specific fluorochrome-conjugated monoclonal antibodies. Altogether, it is important to point out that the specific detection/visualization is necessary to confirm direct transfer of specific proteins from donor to acceptor cell, identified first by high throughput techniques.

## Can Tunneling Nanotubes Mediate Intercellular Protein Transfer?

### Tunneling Nanotubes as a Mechanism of Intercellular Protein Transfer

Tunneling nanotubes (TNTs) are membrane-bound intercellular conduits enabling the transport of various cellular components directly from cell to cell (Rustom et al., 2004). TNTs were shown to enable the transfer of endocytic vesicles, lysosomes and mitochondria as well as membrane-bound proteins (Mittal et al., 2019). They can also be highjacked for direct intercellular viral spread or can transfer misfolded proteins, leading to the propagation of prion diseases and neurodegenerative diseases (Mittal et al., 2019). The involvement of TNTs in the intercellular transfer of membrane-bound proteins has been reported so far—examples will be cited in the following parts of this review. Although, the TNT-mediated transfer of cytosolic proteins, is much less studied, TNTs impede the transfer of small cytosolic molecules such as calcein or GFP (Rustom et al., 2004). Nevertheless, the TNTs formation and protein transfer mechanisms are still not fully known, including the observation that both, open- and closed-ended TNTs exist. Their specific and tight regulation allowing for intercellular transfer is still a matter of debate.

### Possible Mechanisms and Regulators of Tunneling Nanotubes Formation and Function

The exact mechanism of tunneling nanotubes formation still remains not fully known and is likely to depend on the cell type and microenvironment. Resent research suggests two main mechanisms by which cells can form TNTs: filopodial interplay and cell dislodgement. In the first case, when filopodia protrudes from one cell and elongates until it encounters the other one, the conversion towards TNT occurs (Rustom et al., 2004; Bukoreshtliev et al., 2009). When cells stay in contact for a given period of time, then move apart from each other while remaining connected through a thin membranous structure, TNT is formed by a cell dislodgement mechanism (Önfelt et al., 2004; Sowinski et al., 2008, 2011). It is worth noting that these two mechanisms can occur simultaneously, or change dependently on the conditions (Rustom et al., 2004; Wang et al., 2012).

Biran et al. (2015) showed that at least some part of IPT is independent on protein abundance in donor cells and therefore IPT can be a regulated process (Biran et al., 2015). Moreover, the screen of regulators of codeIT, including TNT-mediated transfer, revealed several proteins involved in the regulation of this process (Frei et al., 2015). Myo10 was identified as one of the regulators. As it localises mainly to the tips of cell protrusions, it suggests that those structures are involved. The truncation mutant of Myo10, lacking the motor domain, inhibited transfer only when expressed in donor cells. Cdc42 is another codeIT/TNTs regulator, probably located upstream, as it was not transferred between cells, in contrast to all other regulatory molecules. The Cdc42 knockdown inhibited protein transfer, whereas overexpression of two different mutants of Cdc42 revealed that a cycling between active and inactive forms of Cdc42 is indispensable for this process. Finally, several Rab proteins described to be localised to early, recycling, and tubular endosomes, were found to regulate intercellular protein transfer. The activity of Rab11a, a regulator of recycling endosomes, was important for the process both in donors and acceptors. Its role might be the delivery of cell-cell adhesion molecules to the plasma membrane to enable formation of a tight contact between cells. Moreover, authors proposed several possible roles of Rab35, a known marker of tubular endosomes: determination of sites of F-actin polymerisation, initiation of formation of nanotube precursors or the cadherin-mediated anchoring of nanotubes to target cells. Finally, knockdown of the early endosomal marker Rab5a or EEA1, a known Rab5a-effector, reduced or enhanced transfer, respectively. The list of other identified regulators includes Rab7a, Rab8a and Myo5c (Frei et al., 2015). Altogether those data indicate that mechanisms regulating formation of membrane protrusions are involved in TNTs formation, however this may not be directly associated with the transport of proteins, as only open TNTs are able to finalize the transfer process.

Additional experiments with the transfer of EGFP-tagged regulators showed that all codeIT regulators, except for Cdc42, transferred themselves. This indicates that DiD-labelled vesicles originate from intercellular membranes and the endocytic pathway (Frei et al., 2015). These discoveries led to the model of intercellular protein transfer as a process dependent on F-actin-rich protrusions, positive for Myo10 and regulated by Cdc42, whereas the membrane material is delivered by the endosomal pathway (Frei et al., 2015). The above-described discoveries suggest that TNTs might offer the possible mechanism for contact-dependent intercellular transfer between non-immune cells. Moreover, cytosolic proteins might be transferred enclosed within endosomes, which were identified as the compartment transferred between cells. We also implemented this assumption and used trans-SILAC technique to identify proteins transferred between stromal and leukemic cells within DiD-labelled cellular vesicles. Based on our results, we propose that the intercellular transfer of proteins within these vesicles is an active and tightly regulated process (Kolba et al., 2019).

## TNT-Mediated Transfer of Proteins in Cancer

**FIGURE 2:**
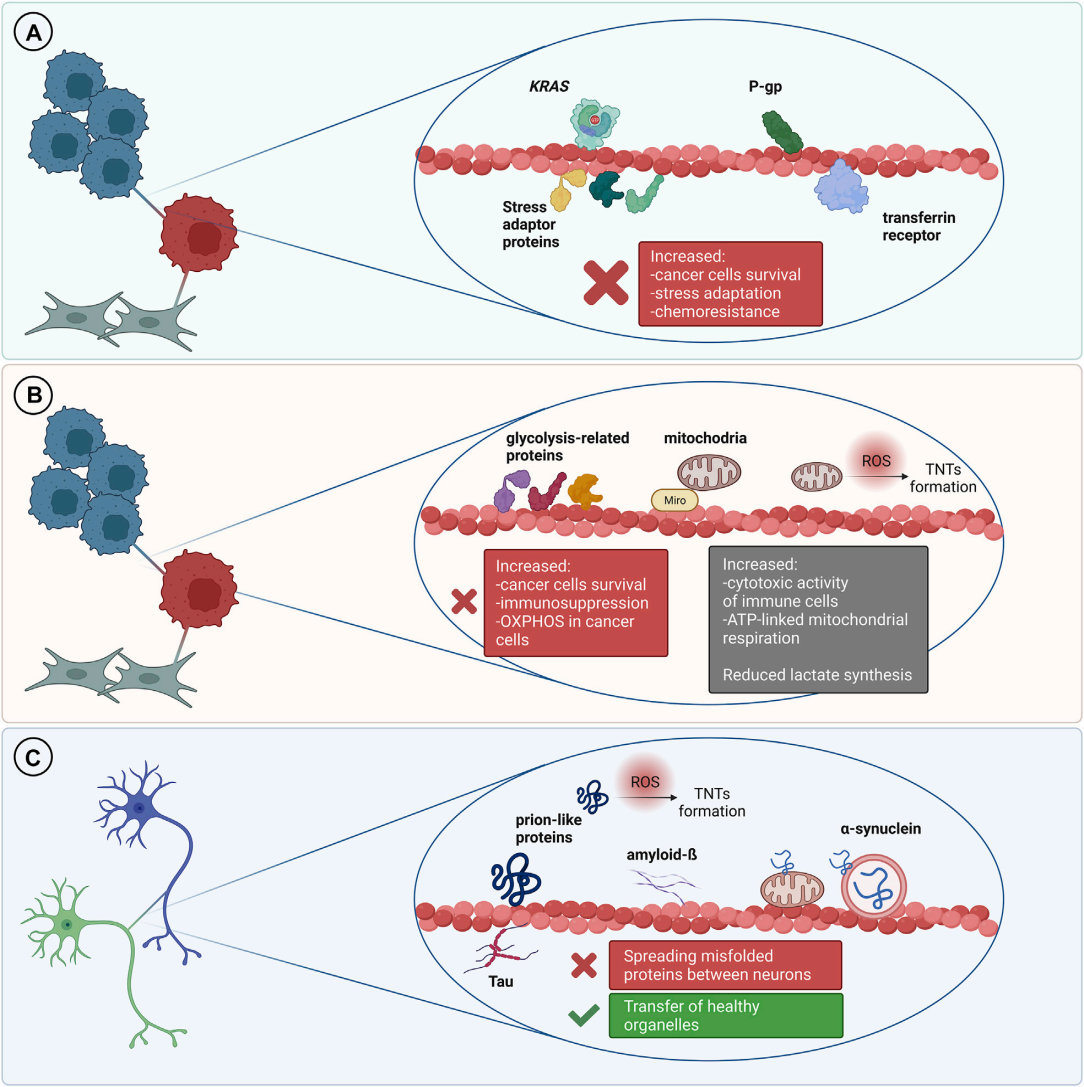
TNT-mediated transfer of various proteins and its outcomes. **(A)** In the cancer microenvironment it is usually associated with cancer progression and increased chemoresistance. **(B)** Transfer of metabolic-related proteins and mitochondria may result in increased immunosuppression but it can also be a cause of increased cytotoxic activity of immune cells. Transfer of mitochondria to cancer cells is often associated with increased chemoresistance and cancer progression. **(C)** TNT can mediate transfer of misfolded proteins between cells in the nervous system which leads to progression of neurodegenerative disorders. On the other hand, TNT can be a route for the transmission of healthy organelles as a rescue mechanism.

An ever-increasing body of literature shows that different types of proteins can be transferred via tunneling nanotubes in the cancer microenvironment (Figure 2). This phenomenon concerns both, membrane and cytosolic proteins, which belong to different functional groups, partially described below. Several methods are reported to address this issue, mainly live cell imaging, time-lapse video microscopy and immunofluorescence.

### Stress Adaptation

Kretschmer et al. showed that androgen receptor blockade and metabolic stress result in induction of TNTs formation between stressed and unstressed prostate cancer cells (PCa) as well as between prostate cancer cells and osteoblasts. They identified three stress adaptor proteins: clusterin (CLU), YB-1 and Hsp27 which localize within TNTs formed between prostate cancer cells (PCa) (Kretschmer et al., 2019). Proximity Ligation Assay further supported these observations. Importantly, authors did not observe stress granules within TNTs, indicating that such structures are not transferred between cells, at least in this model. Using live imaging and fluorescently labelled proteins they confirmed that clusterin is transported bi-directionally *via* TNTs. Moreover, silencing CLU and YB-1 in PCa cells significantly decreased TNTs formation under stress conditions. What is more, it was pointed out that there is a possible regulation that relied upon PI3K pathway in TNTs formation after androgen receptor blockade. Disruption of TNTs formation reduced prostate cancer cells’ survival when treated with androgen deprivation. This indicates a possible role of TNT-mediated transport in the adaptation of prostate cancer cells to stress and cell survival.

### Transfer of Pro-Oncogenic Proteins

It was reported that a mutated form of KRAS protein, involved in development of cancer and chemoresistance (Misale et al., 2012), can be transferred *via* TNTs between colorectal cancer cells (CRC) (Desir et al., 2019). Moreover, CRC harbouring mutant *KRAS* variant (*KRAS G13D*) formed more TNTs than CRC cells with wild-type *KRAS*. GFP-tagged mutant *KRAS* transferred via TNTs from CRC LOVO cells (expressing mutant *KRAS*) to HCT-8 cells (wild-type *KRAS*) was confirmed by fluorescence microscopy and fluorescence recovery after bleaching (FRAP) experiments. Furthermore, co-culture of HCT-8 cells transfected with mutant KRAS with non-transfected HCT-8 cells not only promoted TNTs formation but also proved that mutant *KRAS* can be transported through them, which was presented using fluorescence time-lapse microscopy. Additionally, the transfer of GFP-tagged mutant *KRAS* to wild-type *KRAS* CRC cells was also confirmed by flow cytometry. Moreover, co-culture of wild-type CRC cells with cells harbouring a mutant version of *KRAS,* increased phosphorylation of ERK when compared to wild-type CRC cells cultured alone. This observation indicates a possible role of transferred *KRAS* mutant in inducing ERK activation. This allows to conclude that mutant *KRAS* increases the cross-talk between CRC cells and can be transferred horizontally via tunneling nanotubes.

TNT-mediated transfer of another member of Ras superfamily—H-Ras was shown by Rainy and co-workers. This small GTPase localizes to the inner plasma membrane and can be transferred via TNTs from B cells to T cells (Rainy et al., 2013). This phenomenon was diminished after inhibition of actin polymerization and separation of co-cultured cells with insert which indicated the contact-dependent character of such transport. Researchers applied optical tweezers and 4D spinning disk confocal microscopy to observe TNTs formation between B lymphoblastoid cells and Jurkat cells. GFP-labelled H-Ras protein was transferred through TNTs and GFP-labelled membrane patches segregated from the TNTs were present on the acceptor cells. Authors applied FRAP technique to measure the diffusion of GFP-H-RasG12V both in the TNT and in the membrane patches present on acceptor cells. While photobleaching of GFP-positive regions of TNTs resulted in quick fluorescence recovery, photobleaching of GFP-rich membrane patches present on acceptor cells did not result in fluorescence recovery which confirmed TNT-mediated transport of GFP membrane patches to Jurkat cells. Moreover, labelling of Jurkat cells with an anti-CD86 antibody that specifically recognizes the extracellular domain of CD86—a transmembrane B-cell marker, confirmed that transferred membrane patches maintain their in-out orientation in acceptor T cells. What is more, using specific mutants of H-Ras with prominent cytosolic localization, authors showed that plasma membrane localization of H-Ras is essential for its transfer through tunneling nanotubes.

### Transfer of Cancer Stem Cell Markers

CD133 protein, widely used as a marker for cancer stem cell isolation, was detected within tunnelling nanotubes formed between human primary CD34^+^ hematopoietic progenitors and KG1a—acute myeloid leukemia cells (Reichert et al., 2016). Authors applied time-lapse video microscopy to observe the movement of CD133-GFP puncta along TNTs. They did not report retrograde transport to the donor cells, however, local accumulation of CD133-GFP protein was presented. Reichert et al. suggested that these aggregates could not cross junctional complexes between KG1a and CD34^+^ HPSC cells. To determine if CD133 transfer occurs at the plasma membrane or *via* cytoplasmic structures, they stained live GFP-CD133 KG1a cells with fluorochrome-coupled anti-CD133 antibody. These experiments showed that CD133 transport occurs mainly *via* cell surface (Reichert et al., 2016).

### Proteins Involved in Drug Resistance

Additionally, the well-known drug transporter, P-gp, was reported to be transferred within TNTs between cancer cells. This phenomenon was shown in cultured breast cancer MCF-7 cells by Pasquier and co-workers (Pasquier et al., 2012), where TNTs, together with microparticles, mediated transfer of P-gp between MCF-7 cells. This led to extragenetic emergence of multidrug resistance in a drug-sensitive population of breast cancer cells. First, using flow cytometry, they confirmed that in the co-culture of sensitive and drug-resistant MCF-7 cells, the level of P-gp protein increases with culturing time. Moreover, multidrug resistance activity of co-cultured cells increased, which was shown in the drug efflux assay with calcein AM. Both events were contact-dependent. However, the data was acquired from a mixed population of sensitive and resistant cells, not from single subpopulations. In these co-cultures TNTs were presented never earlier than after 3 days of incubation. Authors labelled sensitive MCF-7 cells with Cell Tracker Violet (to stain cytoplasm) and stained co-culture with WGA (to stain cell membranes) with additional staining with anti-P-gp antibody. Analyses of fixed specimens revealed co-localization of these three dyes and the presence of P-gp enriched “bridges” between MCF-7 resistant and sensitive cells.

### Transfer of Proteins Involved in Tunneling Nanotubes Formation

As already mentioned, proteins involved in tunneling nanotubes formation can be transferred between cells *via* TNTs (D’Aloia et al., 2021). Burtey et al. applied high resolution 4-dimensional confocal microscopy to demonstrate that transferrin receptor (Tf-R) is transferred between HeLa cells. Moreover, Rab8 small GTPase was co-transferred with Tf-R *via* TNTs to acceptor cells (Burtey et al., 2015), and transferrin receptor was detected in vesicular structures visible along tunneling nanotubes. After inhibition of clathrin-mediated endocytosis, transfer of Tf-R decreased, which indicates that endocytosis of the transferrin receptor is required for its TNT-mediated transfer. Similar experiments with GFP-tagged DN mutant of Rab8 co-expressed with Tf-RmCherry demonstrated that Rab8 is also crucial for transfer of Tf-R. Also in the culture of 5637 cells (bladder cancer cells), live imaging microscopy data showed that fluorescently tagged RalA GTPase, which is well-known for its role in TNTs development (Hase et al., 2009), is transported between these cells through TNTs. Another protein significant in TNTs development—LST1 (Schiller et al., 2012) was also effectively transported in this way. It is worth mentioning that both proteins interact with RalGPS2, which was not detected within TNTs but plays a major role in the molecular machinery underlying TNTs formation between bladder cancer cells presented by authors. Interesting observations from Schiller and colleagues (Schiller et al., 2013) showed that transmembrane, but not soluble, HLA-EGFP protein can be transferred between HeLa cells, and inhibition of actin polymerization diminished transfer rate and overexpression of LST1, considered as TNTs formation regulator. All of the data described above is summarized in Table 1.

**TABLE 1:** Transfer of proteins through TNTs in cancer.

Cell Type	Transferred proteins	Protein localization	Methods	References
Prostate cancer cells (PC3, LNCaP)	stress adaptor proteins: CLU, YB-1, Hsp27	CLU- cytosol, HSP27 plasma membrane and cytosol, YB-1 plasma membrane, cytosol, ER, vesicles	Immunofluorescence	Kretschmer et al. (2019)
colorectoral cancer cells (HCT-8, LOVO)	mutant KRAS G12D	Plasma membrane	Fluorescence microscopy, FRAP, time-lapse microscopy, flow cytometry	Desir et al. (2019)
B721.221 (B cells), Jurkat (T cells)	H-Ras	Plasma membrane	Confocal microscopy, FRAP	Rainy et al. (2013)
MCF-7 (breast cancer cells)	P-gp	Plasma membrane	Live cell microscopy, immunofluorescence, flow cytometry	Pasquier et al. (2012)
HeLa	transferrin receptor	Plasma membrane	High resolution 4D confocal microscopy	Burtey et al. (2015)
5637 (bladder cancer cells)	RalA, LST1	Plasma membrane	Live cell imaging	D’Aloia et al. (2021)
HeLa	MHC I	Plasma membrane	Confocal microscopy	Schiller et al. (2013)

FRAP, fluorescence recovery after bleaching.

## Transfer of Metabolic-Related Proteins

An increasing number of evidence demonstrates that IPT is also frequently associated with metabolic adaptation of the recipient cells. Such phenomenon was recently investigated in the context of cancer progression, immune response, drug resistance and tissue rejuvenation (Hekmatshoar et al., 2018; Mittal et al., 2019) (Figure 2). In tumors, metabolism of malignant cells is characterized by the Warburg effect—an increased glucose uptake and lactate fermentation, even in the presence of oxygen and fully functional mitochondria. This leads to mitochondria reprograming to supply anabolic pathways to and support rapid proliferation (DeBerardinis and Chandel, 2016). Alterations in mtDNA, including mutations, depletions or reduced copy numbers, are common hallmarks of cancer, including response to chemotherapy, therefore, restoration of the mitochondrial function is important for development of the resistance and cancer progression (Guerra et al., 2017).

Soon after TNTs were discovered, mitochondria were identified among TNT cargos (Spees et al., 2006; Önfelt et al., 2006).

Most of the 1,100 different proteins that build human mitochondria (Rath et al., 2021) is synthesized in the cytosol as protein precursors (Neupert and Herrmann, 2007), which are unstable and can compromise the cellular protein homeostasis (Liu et al., 2019; Nowicka et al., 2021). Thus, the intercellular transfer of the whole organelles, potentially provides the immediate effect without disturbing cytosolic proteostasis of the recipient cells. Even though, there are other pathways that allow mitochondrial uptake by the cell, including extracellular vesicles, free-mitochondria uptake, cell fusion and gap-junctions, TNTs have recently gained most of the attention due to its targeted and inducible characteristics (reviewed in Yan et al., 2021; Zampieri et al., 2021). TNTs formation was shown to be enhanced by starvation, reactive oxygen species or chemotherapeutic drugs (Zhu et al., 2005; Desir et al., 2019). At the molecular level, Lu and colleagues found that number of TNTs positively correlated with metabolism-related Akt-mTOR signaling in malignant urothelial T24 cells (Lu et al., 2017). Another study showed that multiple myeloma cells utilize plasma membrane NADPH oxidase 2 (NOX2) to generate ROS and stimulate mesenchymal stem cells (MSCs) for TNTs formation and mitochondria transfer (Marlein et al., 2017). What is more, the process of mitochondria motility through TNTs is based on cytoskeletal filaments and is regulated by a calcium-sensitive adaptor protein—Miro1 (mitochondrial Rho GTPase 1, synonym: Rhot1), which ties mitochondrion with motor complex (Fransson et al., 2006; Ahmad et al., 2014). Remarkably, phosphorylation of Miro1 protein causes dissipation of the damaged mitochondrion from the motor protein complex and induce autophagy (Narendra et al., 2008; Safiulina et al., 2019). Despite the molecular mechanism of TNT function remains elusive, it seems that TNT-mediated communication between cells is precisely regulated and delivers mitochondria of a good quality.

### Importance in Cancer

The first evidence of functional horizontal mitochondrial transfer was presented on human MSCs and skin fibroblasts, which rescued mitochondria function in mtDNA-depleted lung carcinoma cell line—A549 rho^0^ cells. After direct co-culture, A549 cells re-established the level of intracellular ATP and oxygen consumption with simultaneous decrease of extracellular lactate and ROS production. This indicated a recovered respiratory function and oxidative metabolism. The examination of mitochondrial and DNA polymorphisms in the rescued clones confirmed the successful horizontal mitochondria transfer between cells, as well as excluded the role of cell fusion in this process (Spees et al., 2006). Even though the direct evidence for TNT activity in this work was not provided, the co-localization of mitochondria with TNT was documented by microscopic imaging in numerous following studies. Till now, mitochondria transfer to cancer cells *via* TNTs was confirmed in different *in vitro* and *ex vivo* experimental set-ups including lung (Ahmad et al., 2014), breast (Pasquier et al., 2013; Tan et al., 2015), ovarian (Pasquier et al., 2013), bladder (Lu et al., 2017), brain (Pinto et al., 2021) and blood cancers (Moschoi et al., 2016; Marlein et al., 2017; Wang et al., 2018; Burt et al., 2019; Kolba et al., 2019). Reviewed in Vignais et al. (2017), Hekmatshoar et al. (2018), and Yan et al. (2021).

### Effect on Immunometabolism

In many cases, modification of cell bioenergetics by increased OXPHOS at the expense of glycolysis was observed. Recently, Saha and colleagues discovered also that cancer cells utilize TNTs to hijack mitochondria from immune cells to suppress their cytotoxic activity. By combining the transient (MitoTracker, dye-based) and stable (genetic-based) fluorescent labelling of mitochondria with microscopic and flow cytometry detection techniques, authors documented that mitochondria are transferred unidirectionally from T cells (CD3^+^/CD3^+^ CD8^+^/NKT) to breast cancer cells *via* TNTs. The possibility of indirect mitochondria transfer *via* extracellular vesicles was excluded, as it did not occur in the transwell co-culture set-up. Mitochondria hijacking resulted in increased basal and spare mitochondrial respiration as well as enhanced proliferation of cancer cells. On the other hand, organelle outflow from T cells resulted in a reduced aerobic respiration and decreased number of cells (Saha et al., 2022). In the immune cells, the cytotoxic activity and metabolism are correlated (Mathis and Shoelson, 2011; Klein Geltink et al., 2018). Thus, TNT-mediated transfer provides both metabolic and immunosuppressive benefits for cancer growth (Saha et al., 2022). The effect of TNT-mediated communication on immunometabolism was also observed in the context of non-malignant cells. Specifically, macrophages were shown to obtain mitochondria from MSCs *via* TNTs in *in vitro* and *in vivo* models of *Escherichia coli pneumonia*. As expected, mitochondria transfer led to significant and strong increase in basal and ATP-linked mitochondrial respiration in macrophages, as measured by enhanced oxygen consumption rate and reduced lactate synthesis. Simultaneously, co-culture with MSCs enhanced their bacteria phagocytic capacity. Both respiratory and phagocytic effect was partially inhibited by pre-treatment of MSCs with cytochalasin B—an actin polymerization inhibitor, which inhibits TNT-mediated communication (Jackson et al., 2016). Incomplete inhibitory effect can be explained by the fact that transfer of functional mitochondria between those cells can also be governed by extracellular vesicles that lead to the same phenotypic changes (Morrison et al., 2017).

Immunometabolic effect of mitochondria transfer was also observed in active Th17 cells, which generate most of their ATP in glycolysis (Kono et al., 2018). Notably, Luz-Crawford and colleagues have found that Th17 cells among other primary T cell subpopulations, are most efficient in taking up mitochondria during direct co-culture with bone marrow MSCs. Mitochondria acceptors showed elevated aerobic respiration and reduced production of pro-inflammatory IL-17. Moreover, authors discovered that a higher percentage of Th17 effector memory cells that received mitochondria acquire a regulatory T cell phenotype, compared to their counterparts which did not received mitochondria. This indicates that Th17 pro-inflamatory activity is negatively regulated by MSC cells through TNT-mediated IPT. Furthermore, co-culture of Th17 cells with MSCs derived from patients with rheumatoid arthritis, in contrast to healthy MSCs, showed decreased mitochondria transfer, meaning that altered Th17 regulation through mitochondria uptake can be involved in pathogenesis of rheumatoid arthritis (Luz-Crawford et al., 2019). Cdc42 protein is also recognized as a central regulator of Th17/Treg balance and determines the pathogenic phenotype of Th17 cells, characterized by upregulated glycolysis. Intriguingly, T-cells obtained from mice with Cdc42-deficiency manifested increased susceptibility to intestinal damage and pathogenic inflammation (Kalim et al., 2018), therefore supporting the hypothesis postulated by Luz-Crawford et al.

## TNT-Mediated Self-Infection of Neurons in Neurodegenerative Disorders

It is well established that spreading of amyloidogenic proteins occurs through secretory mechanisms, including exosomes, thus contributing to exacerbation of neurodegenerative diseases (Lee et al., 2011). However, growing evidence indicates that TNT-mediated protein transfer also plays a role in propagating neurodegenerative pathologies (Figure 2.). It was demonstrated that a variety of misfolded proteins, including tau (Abounit et al., 2016; Tardivel et al., 2016; Chastagner et al., 2020), α-synuclein (Dilsizoglu Senol et al., 2021), prions (Gousset et al., 2009; Zhu et al., 2015) and mutant huntingtin (Costanzo et al., 2013; Tang, 2018), can be transferred through TNTs in neuronal cells. Importantly, not only neurons’ infection, but also their exposure to amyloidogenic proteins supports intercellular transfer by increasing the number of TNT connections between cells (Costanzo et al., 2013; Zhu et al., 2015; Abounit et al., 2016; Tardivel et al., 2016). Neurodegenerative diseases and accumulation of cytotoxic protein assemblies are associated with oxidative stress. Therefore, it has been proposed that the prion-like proteins might contribute to generation of reactive oxygen species, which in turn stimulate TNTs formation as a stress response mechanism (Abounit et al., 2016; Victoria and Zurzolo, 2017). Propagation of aggregates can differ depending on their origin. However, both endogenously formed (Chastagner et al., 2020) and internalized (Abounit et al., 2016) tau aggregates were found within TNTs formed between neurons. Misfolded proteins can be transferred through TNTs, either inside the vesicles or as protein aggregates associated with organelles or proteins. In CAD (Cath.-a-differentiated) cells, the prions responsible for transmissible spongiform encephalopathies and Parkinson’s Disease-causing α-synuclein aggregates can be found within TNTs in endolysosomal vesicles and lysosomes, respectively. Both PrPSc and α-synuclein were found to colocalize with endosomal and lysosomal markers, confirming the transport (Zhu et al., 2015; Dilsizoglu Senol et al., 2021). Moreover, α-synuclein fibrils can damage lysosome structure and promote peripheral redistribution of α-synuclein-bearing lysosomes in neuronal cells, leading to enhanced α-synuclein transfer *via* TNTs to neighbouring cells (Dilsizoglu Senol et al., 2021). Studying TNTs is currently mostly based on imaging methods, such as fluorescence microscopy (FM). However, due to low-resolution of FM it is often challenging to obtain desirable data. Employing more advanced imaging tools, such as super-resolution (SR) microscopy or combining different approaches, seems to overcome this issue. For instance, structured illumination microscopy (SIM) demonstrated α-synuclein localization both inside lysosomes and at their membrane, whilst correlative light-electron microscopy enabled identifying α-synuclein positive lysosomes by FM and studying their corresponding size and morphology by electron microscopy (EM) (Dilsizoglu Senol et al., 2021). Mitochondria-associated TNT-mediated α-synuclein transfer was also reported. SR microscopy data obtained *via* stimulated emission depletion microscopy (STED) presented α-synuclein bound to the mitochondrial outer membrane, both in cytoplasm and within TNTs of connected cells (Valdinocci et al., 2021). Mutant huntingtin and other poly-Q expanded proteins can be selectively transported by Rhes protein primarily in lysosomal vesicles within TNT-like Rhes-induced protrusions in murine striatal neuronal cells (Sharma and Subramaniam, 2019). Finally, using a combination of live imaging, light- and cryo-electron microscopy approaches allowed studying TNTs’ complex structure at nanometre resolution in murine CAD and human neuroblastoma SH-SY5Y model cell lines. It was reported that single TNTs observed by FM consist of a bundle of individual TNTs (iTNTs) held together and stabilized by N-Cadherin. Each iTNT is filled with a parallel actin bundle, which enables cargo transport, presumably involving myosin motor proteins. Additionally, correlative focused-ion beam SEM (FIB-SEM) demonstrated that TNTs can be open on both ends (Sartori-Rupp et al., 2019). Electrophysiology assay can be used to assess electrical properties and type of TNTs, whether TNTs allow transport of cargo or not (Dieriks et al., 2017). However, to determine the efficacy of TNT-mediated cargo transfer, the flow cytometry methodology is often used in studying neurodegenerative disorders (Costanzo et al., 2013; Sharma and Subramaniam, 2019; Chastagner et al., 2020). To confirm that protein transfer occurs through contact-dependent mechanism, acceptor cells can be incubated in the supernatant of donor cells or both cell types can be physically separated, i.e., by filters, and then analyzed by flow cytometry (Costanzo et al., 2013; Sharma and Subramaniam, 2019). Noteworthy, non-neuronal cells might also contribute to spreading of misfolded proteins in the brain. Human pericytes (Dieriks et al., 2017) and astrocytes (Rostami et al., 2017) frequently form TNTs and transfer α-synuclein aggregates. TNTs can be formed in homotypic and heterotypic co-cultures (Chastagner et al., 2020; Dilsizoglu Senol et al., 2021). Chastagner et al. (2020) identified tau fibrils TNT-mediated transfer from neurons to astrocytes and Gousset et al. (2009) demonstrated that dendritic cells are capable of transporting prions to CNS (central nervous system) *via* TNTs, thus emphasizing the variety of cells contributing to misfolded proteins spreading via direct cell-cell connections. Importantly, a recent study presented evidence for a TNT-mediated green fluorescent protein transport from astrocytes to neurons *in vivo*, indicating a possible similar mechanism for prion-like proteins propagation in the brain (Chen and Cao, 2021). Interestingly, emerging evidence demonstrates aggregates degradation supported by TNT-mediated protein distribution. Microglia overloaded with α-synuclein can transfer cytotoxic proteins to healthy microglia, thus supporting aggregates degradation, inflammation attenuation and overall decrease of cytotoxicity, improving microglial survival *in vitro* and *in vivo* (Scheiblich et al., 2021). Similarly, TNT-mediated crosstalk between astrocytes and microglia increases α-synuclein and amyloid-ß aggregates degradation. Microglia, however, are more efficient in aggregates clearance than astrocytes (Rostami et al., 2021). Altogether it seems that in such context, the microenvironment can utilize the TNT network and microglia connections to protect neurons from aggregate-mediated cytotoxicity. Some studies reported that acceptor cells which received a pathological protein send out healthy lysosomes (Dilsizoglu Senol et al., 2021) or mitochondria (Rostami et al., 2017; Scheiblich et al., 2021) in return, possibly as a TNT-mediated rescue mechanism for infected cells. Although there are emerging reports on the presence of TNTs in CNS (Alarcon-Martinez et al., 2020) and TNT-mediated intercellular protein transfer in the brain (Chen and Cao, 2021), it remains challenging to study TNTs in neurodegenerative diseases *in vivo*. All of the data described in this chapter is summarized in Table 2.

**TABLE 2:** Transfer of proteins through TNTs in neurodegenerative disorders.

Cell type	Transferred proteins	Methods	Additional informations	References
CAD; HeLa	α-synuclein	Confocal microscopy, Co-localization studies, SR SIM, live spinning-disk microscopy	α-syn aggregates transfer inside lysosomes; transfer of healthy lysosomes to damaged cells	Dilsizoglu Senol et al. (2021)
		CLEM		
SH-SY5Y; human post-mortem brain pericytes	α-synuclein	Confocal microscopy, SEM, electrophysiology		Dieriks et al. (2017)
1321N1; differentiated microglia-like THP1;	α-synuclein	Confocal microscopy, STED	α-syn aggregates associated with mitochondrial outer membrane	Valdinocci et al. (2021)
SH-SY5Y				
Human ESC-derived astrocytes	α-synuclein	Confocal microscopy, TEM	Transfer of healthy mitochondria to damaged cells	Rostami et al. (2017)
Mouse primary microglia;	α-synuclein	Confocal microscopy, *in vivo* 2-photon microscopy, flow cytometry	α-syn aggregates redistribution and degradation; transfer of healthy mitochondria to α-syn-overloaded microglia	Scheiblich et al. (2021)
Human monocyte-derived microglia;				
Mouse organotypic slice culture (OSCs);				
Human post-mortem brain sections				
Human iPSC-derived astrocytes and microglia	α-synuclein amyloid- ß	Confocal microscopy	Microglia degrade aggregates more efficiently	Rostami et al. (2021)
CAD; HeLa	tau	Epifluorescence microscopy		Abounit et al. (2016)
CAD; rat primary neurons	tau	Confocal microscopy, spinning-disk confocal microscopy, co-localization, TEM	Soluble and fibrillar tau co-localizes with actin	Tardivel et al. (2016)
CAD;SH-SY5Y; mouse primary neurons	tau	Confocal microscopy, flow cytometry, IncuCyte	Endogenously formed tau aggregates transfer	Chastagner et al. (2020)

CAD;Mouse primary cerebellar granule neurons	mHtt	Wide-field fluorescence microscopy, flow cytometry		Costanzo et al. (2013)

Mouse striatal neuronal cells (cell line and primary cells)	mHtt	Flow cytometry, confocal microscopy, SEM, TEM	“Rhes tunnels”;	Sharma and Subramaniam (2019)
			Rhes protein-associated transfer	
CAD; Mouse primary cerebellar granule neurons;	PrPSc	Confocal microscopy, spinning-disk confocal microscopy		Gousset et al. (2009)
Mouse primary bone-marrow-derived dendritic cells;				
Mouse primary embryonic hippocampal neurons				
CAD	PrPSc	Co-localization		Zhu et al. (2015)
		Confocal microscopy, flow cytometry		

SR SIM, super resolution structures illumination microscopy; CLEM, correlative light-electron microscopy; SEM, scanning electron microscopy; STED, stimulated emission depletion microscopy; TEM, transmission electron microscopy, IncuCyte—real-time live-cell imaging and analysis system.

## Concluding Remarks

Accumulating evidence has indicated the important role of tunneling nanotubes in the intercellular protein transfer between distant cells, as well as other molecules or organelles involved in many pathological conditions (mainly cancer and neurodegenerative disorders). In this review we focused on the one hand, on different examples of intercellular and TNT-mediated intercellular protein transfer between distant cells of different origin, and on the other hand, on methods used for studying IPT, mainly with regards to transfer *via* TNTs. Despite the advanced approaches used to investigate the intercellular protein transfer, like trans-SILAC or codeIT, and great development of other techniques used for hit validation, particularly microscopy, further efforts on this topic are required. Considering particular fragility of TNTs structure, it seems that in many cases the greatest remaining challenge is to transfer the results obtained using *in vitro* cellular models into *in vivo* conditions.
